# Obesogenic and leptogenic medication utilization in people with obesity: Is there a prescription for improvement?

**DOI:** 10.1016/j.lana.2022.100259

**Published:** 2022-04-22

**Authors:** Eduardo Grunvald

**Affiliations:** a Division of General Internal Medicine, Department of Medicine, University of California San Diego, La Jolla, CA, USA; b Center for Advanced Weight Management, Bariatric and Metabolic Institute, Department of Surgery, University of California San Diego, 4303 La Jolla Village Drive, Suite 2110, San Diego, CA, USA

The obesity pandemic shows no signs of waning. Successful efforts for prevention and reversal of excess weight on a population level have remained elusive. Obesity is a complex disease with multiple etiological factors, including genetic, hormonal, metabolic, environmental, economic, psychosocial, and cultural contributors, with great variability in their relative effects between individuals. Another potential influence is the use of obesogenic medications to treat a myriad of symptoms and chronic diseases.^1^ Iatrogenic weight gain is often underappreciated when managing patients in the clinical setting. Evidence for the prevalence of obesogenic drug utilization in the general population is scarce. Conversely, anti-obesity medications (AOMs) have been prescribed at very low levels.^2^

In this issue of *The Lancet Regional Health - Americas*, Lyu et al.^3^ used logistic regression models on data from the United States National Health and Nutrition Examination Survey (US NHANES) spanning 2009–2018 to evaluate the association between socioeconomic status (SES) and use of both obesogenic drugs and AOMs. They analyzed a composite unadjusted model and models adjusted for covariates such as age, sex, race, medication use, and comorbidities. Consistent with epidemiological reports, their analysis confirmed a significant inverse relationship between SES and body mass index. Almost 40% of individuals in their population were prescribed obesogenic agents. Subjects with lower SES were more likely to use medications that can cause weight gain.

Previous work using data from NHANES, from a longer, earlier period of observation, also showed significant prevalence of obesogenic drug use with increasing prescriptions over time.^4^ Similar to the work by Lyu et al., the most common agents were anticonvulsants, antidepressants, antipsychotics and beta-blockers. Should prescribers care? The previously accepted paradigm of weight control through simple voluntary balance of calorie consumption and energy expenditure is outdated. We now understand, based on years of scientific data, that propensity for weight gain, defense of energy stores, and resistance to weight loss and maintenance are all based on complex internal biological and external environmental factors. Given that many, if not most, of these challenges are not easily modifiable, clinicians should strive to avoid or remove one of the obstacles - obesogenic medications - whenever possible. Rather than considering mean weight gain attributable to an individual agent, prescribers should consider its effect on the physiologic fat mass set point.^5^

This may be easier said than done, however. As noted by Lyu et al., even when non-obesogenic alternatives exist, implementation may be deprioritized by limited awareness when considering weight change potential of pharmaceuticals, pharmacotherapy inertia, administrative and time burden, and cost. The latter possibility may be a critical contributor to their findings. The sub-analysis in the report using anti-diabetes treatment exemplifies the possibility that reduced access and insurance coverage for newer, more costly alternatives places individuals of lower SES at further disadvantage and increased risk for weight gain. In the Look AHEAD study, a large, randomized lifestyle intervention trial in subjects with diabetes, racial minorities and subjects with lower incomes were less likely to be initiated on novel, weight neutral or leptogenic (causing weight loss) anti-diabetic medications.^6^

Moreover, high quality data to guide best practices are lacking. Apovian et al. referenced guidelines published in 2015 recommending less obesogenic options when available and appropriate.^7^ These evidence-based recommendations are necessary and helpful, but there are no large-scale randomized controlled trials specifically comparing medications for weight gain on a long-term basis. Data are also scarce regarding weight loss outcomes, with the exception perhaps for anti-diabetic therapies, after transition from an obesogenic medication to a neutral or leptogenic option. More research is needed in this regard to better inform changes in prescribing practices.

In contrast, there is rapidly increasing evidence for the superior effectiveness and benefits of AOMs compared to lifestyle interventions alone.^8^ Despite this, utilization has remained very low in the US.^2^^,^^9^ Also in line with findings reported by Lyu et al., racial minorities and lower SES - those more affected by obesity - predict lower likelihood of receiving prescriptions for weight loss. Reasons include lack of education and training for treating obesity, negative legacy effect of adverse outcomes with previous drugs, perceived suboptimal efficacy, poor payor reimbursement, and high administrative burden with insurance authorization requirements. Racial/ethnic minorities and those with lower SES also tend to be underinsured. The work by Lyu et al. illuminates that these vulnerable populations are therefore burdened with dual inequities, increased risk of obesity and inferior access to high quality care affecting weight outcomes.

Can we do better? Aside from improving on the obstacles highlighted previously, obesity needs to be accepted as a biological condition. Like any other chronic disease encountered in modern clinical practice, medical professionals should understand the pathophysiologic principles that govern weight regulation and apply evidence-based therapies as equitably as possible. Currently, obesity receives inadequate attention in US medical school curricula.^10^ If prescribing practices are one of many strategies used to combat the obesity epidemic, we need to increase the dose of awareness, education, and evidence-based interventions to help our patients, regardless of their socioeconomic position in society.

## Declaration of interests

EG has received consulting fees from Novo Nordisk, Currax Pharmaceuticals, and Gelesis, Inc. He has also received research support from the Obesity Treatment Foundation (Grant No OTF001), The Litwin IBD Pioneers Program Crohn's and Colitis Foundation, Aardvark Therapeutics, Eli Lilly and Rhythm Pharmaceuticals.
